# Losing sleep influences dietary intake in children: a longitudinal compositional analysis of a randomised crossover trial

**DOI:** 10.1186/s12966-024-01607-5

**Published:** 2024-06-04

**Authors:** Jillian J. Haszard, Rosie Jackson, Silke Morrison, Kim A. Meredith-Jones, Barbara C. Galland, Dean W. Beebe, Dawn E. Elder, Rachael W. Taylor

**Affiliations:** 1 Biostatistics Centre, University of Otago, Dunedin, New Zealand; 2 Department of Medicine, University of Otago, PO Box 56, Dunedin, 9016 New Zealand; 3 Department of Women’s and Children’s Health, University of Otago, Dunedin, New Zealand; 4 Department of Pediatrics, Division of Behavioral Medicine and Clinical Psychology, University of Cincinnati College of Medicine, Cincinnati Children’s Hospital Medical Centre, Cincinnati, OH USA; 5 Department of Paediatrics and Child Health, University of Otago, Wellington, New Zealand

**Keywords:** Obesity, Child, Sleep, Compositional time use, CoDA, Time reallocation, Physical activity, Sedentary behavior, Sedentary time

## Abstract

**Background:**

Although inadequate sleep increases the risk of obesity in children, the mechanisms remain unclear. The aims of this study were to assess how sleep loss influenced dietary intake in children while accounting for corresponding changes in sedentary time and physical activity; and to investigate how changes in time use related to dietary intake.

**Methods:**

A randomized crossover trial in 105 healthy children (8–12 years) with normal sleep (~ 8–11 h/night) compared sleep extension (asked to turn lights off one hour earlier than usual for one week) and sleep restriction (turn lights off one hour later) conditions, separated by a washout week. 24-h time-use behaviors (sleep, wake after sleep onset, physical activity, sedentary time) were assessed using waist-worn actigraphy and dietary intake using two multiple-pass diet recalls during each intervention week. Longitudinal compositional analysis was undertaken with mixed effects regression models using isometric log ratios of time use variables as exposures and dietary variables as outcomes, and participant as a random effect.

**Results:**

Eighty three children (10.2 years, 53% female, 62% healthy weight) had 47.9 (SD 30.1) minutes less sleep during the restriction week but were also awake for 8.5 (21.4) minutes less at night. They spent this extra time awake in the day being more sedentary (+ 31 min) and more active (+ 21 min light physical activity, + 4 min MVPA). After adjusting for all changes in 24-h time use, losing 48 min of sleep was associated with consuming significantly more energy (262 kJ, 95% CI:55,470), all of which was from non-core foods (314 kJ; 43, 638). Increases in sedentary time were related to increased energy intake from non-core foods (177 kJ; 25, 329) whereas increases in MVPA were associated with higher intake from core foods (72 kJ; 7,136). Changes in diet were greater in female participants.

**Conclusion:**

Loss of sleep was associated with increased energy intake, especially of non-core foods, independent of changes in sedentary time and physical activity. Interventions focusing on improving sleep may be beneficial for improving dietary intake and weight status in children.

**Trial Registration:**

Australian New Zealand Clinical Trials Registry ANZCTR ACTRN12618001671257, Registered 10th Oct 2018, https://www.anzctr.org.au/Trial/Registration/TrialReview.aspx?id=367587&isReview=true

**Supplementary Information:**

The online version contains supplementary material available at 10.1186/s12966-024-01607-5.

## Background

Short sleep duration has been consistently and strongly related to an increased risk of obesity in children [1, 2]. While the mechanisms remain unclear, they may include changes in behaviors that contribute to energy balance, such as sedentary time, physical activity, and/or dietary intake [3]. Although a considerable body of literature has examined the relationships between individual energy balance behaviours and weight status in children [4–7], it is increasingly recognised that these energy balance behaviours are intrinsically related to each other in many ways [8, 9]. Thus, examining how any one behaviour (e.g. sleep) affects the outcome (e.g. weight), cannot really be undertaken without due consideration of the other interrelated behaviours.

This poses an issue for studies examining sleep, sedentary time, and physical activity as an increase in time spent in one behavior must necessarily decrease time in one or more of the other behaviors, due to the constraints of the 24-h day. This interdependence introduces confounding, so that associations that have not appropriately accounted for the time use composition of the whole day are not accurate—they do not provide a good estimate of the relationship between the specific time use behavior and the health outcome of interest (e.g. sleep duration and adiposity). However, simply including all time use behaviors together in a multivariate regression model in an attempt to adjust for the influence of the other time use behaviors violates the requirement that variables should be independent. Recently a compositional data analysis (CoDA) framework was developed that allows for the estimation of associations between time use components (sleep, sedentary time, light physical activity (LPA), and moderate-to-vigorous physical activity (MVPA)) and health outcomes, while accounting for all other components of the day [10, 11]. Current compositional analyses in children are mostly cross-sectional and suggest that more time asleep and/or in MVPA, and less time sedentary are associated with lower BMI z-score [5, 12–14]. Such cross-sectional analyses can only provide correlational estimates, are still likely to be confounded by other unmeasured variables (such as diet), and offer little insight to how change in sleep actually *influences* outcomes.

Our recent randomised crossover trial showed that when children were mildly sleep deprived they were not less physically active compared to when they were well-rested, suggesting that the mechanism between short sleep and obesity is through diet, rather than lower energy expenditure [15]. This was supported by the result that energy intake significantly increased with sleep loss [16]. However, this increased energy intake may have been because of the increase in physical activity or the increase in sedentary time that occurred with the sleep loss, not because of the sleep loss per se. To determine if sleep loss influences dietary intake independent of the changes in physical activity and sedentary time, longitudinal compositional analyses from intervention studies that have used high quality assessments of both time-use and dietary intake are required. To date, there appear to have been no compositional studies that have explored associations with dietary intake, which is likely to be a key mediator between time use and health. Nor have longitudinal compositional studies appear to have reported the relationship between changes (or ‘reallocations’) in time and the effect on dietary intake.

In this paper, we aim to assess how sleep loss influenced dietary intake in children while accounting for the corresponding changes in sedentary time and physical activity. In addition, we investigate how changes in physical activity and sedentary time that occur when sleep is lost, relate to dietary intake.

## Methods

### Overview

Daily, Rest, Eating, and Activity Monitoring (DREAM) was a mechanistic study aiming to determine the changes in energy balance behaviours that occur during a period of mild sleep deprivation [17, 18]. In this randomized crossover trial, we compared a week of sleep restriction (children asked to go to bed one hour later than their usual bedtime each night), with a week of sleep extension (children asked to go to bed one hour earlier than their usual bedtime each night), separated by a one-week washout. Measures of dietary intake (two 24-h recalls) and 24-h time use (7-day accelerometry) were obtained during each intervention week.

### Subjects

Participants were recruited via social media and word of mouth in Dunedin, New Zealand from October 2018 to March 2020. Children were eligible if they were aged 8–12 years at enrolment, had no underlying medical conditions, took no medications or supplements that could impact sleep or diet, and scored 39 or less on the Sleep Disturbance Scale for Children [19], indicating they were healthy sleepers. To ensure our intervention did not put children in the ‘not recommended’ category for sleep duration (< 7 h or > 12 h) [20], children had to have a parent-reported time in bed each night of 8–11 h. The primary caregiver gave written informed consent and the child written informed assent. Participants (NZ$100) and caregivers ($NZ50) received gift vouchers at the end of the study. DREAM was approved by the University of Otago Human Ethics Committee (Reference # 18/146) and registered with the Australian New Zealand Clinical Trial Registry (ACTRN12618001671257).

### Intervention

Usual bedtime at baseline was determined from a 7-day sleep diary and discussion with parents to ascertain whether these averages reflected ‘usual’ bedtimes. We specifically chose to use sleep extension as the ‘control’ condition, rather than baseline or ‘usual’ sleep to create a clear deprivation state but without causing potential harm to those who may have not had sufficient sleep at baseline. In such participants, a more severe sleep restriction protocol (e.g. delaying bedtime by two hours rather than one hour to create the same potential difference between sleep conditions) could have put some children in the ‘not recommended’ category for sleep duration [20]. Allowing children to extend their ‘usual’ sleep ensured our ‘active’ condition (sleep restriction) could be compared with the ‘inactive’ condition (sleep extension). The intervention was only undertaken during the school term and children had to keep wake time the same as it was at baseline to mirror normal environmental conditions (i.e., getting up for school).

Randomization to the order in which restriction and extension occurred was stratified by gender and age (grouped by 8–10, 11–12 years), generated by the study biostatistician, using random block lengths (Stata 15.1, StataCorp, Texas) in a 1:1 allocation, and uploaded to the Research Electronic Data Capture (REDCap) tools randomization module. Randomization assignment was conducted by a researcher after baseline assessment. While researchers collecting dietary data were aware of participant allocation due to the nature of the study, the researchers entering the dietary and accelerometry data and the biostatistician were blinded to allocation.

Sample size calculations were based on detecting a difference of 500 kJ (using SD of 1619 kJ [14]) in daily energy intake (80% power, α 0.05), requiring 85 children. We aimed to recruit 110 children to allow for 20%-40% drop out given the intensive nature of the study [17].

### Measures at baseline

At baseline, parents completed questions on child age, gender, and ethnicity, maternal education, and area-level deprivation as an indicator of socioeconomic status [21]. Weight (Tanita electronic scales HD351) to the nearest 0.1 kg and height (Wedderburn, Portable Height Rod, WS-HRP) to the nearest 0.5 cm were obtained by trained researchers in duplicate, with children wearing light clothing and no shoes, using standard procedures. Body mass index (BMI) z-scores were calculated based on WHO reference data for 5–19 years old, with normal weight defined as a BMI z-score ≤ 1; overweight as BMI z-score > 1 & ≤ 2; and obese as BMI z-score > 2 [22].

### Measures during each intervention week

24-h time use (sleep, sedentary time, physical activity) was measured over 7 days and nights using an Actigraph (ActiGraph wGT3X-BT, Pensacola, USA), attached to the skin (waterproof) or worn on an elastic belt (not waterproof) on the participant’s right hip for 24 h/day over eight days. The ActiGraph was initialised using Actilife software (Version 9.0.0) to commence at 12:00 AM on the day of the first appointment. Data were cleaned and scored using an automated script developed in MatLab® (MathWorks, Natick, MA,USA)[23, 24]. Sleep onset (child goes to sleep), sleep offset (child wakes), sleep period time (SPT, time between sleep onset and offset), and total sleep time (TST, time between sleep onset and offset, minus any time awake in the night) were determined for each individual night. Non-wear time (at least 20 min of consecutive zeros), and time spent sedentary and in light, moderate and vigorous physical activity were then determined during awake hours only using the Evenson cutpoints [25]. A valid day was defined as at least eight hours of wear-time during awake hours, at least four hours of sleep, and less than two hours of non-wear time while awake. Data were averaged across the week, weighted for weekend days (2/7), and weekdays (5/7), and normalised to 24 h [15].

Dietary intake was assessed by trained research staff on days 2 and 7 of each intervention week using 24-h multiple pass diet recalls (24-h MPDR), supplemented by photos taken by participants of all foods and beverages consumed which acted as memory prompts. Both the child and parent were present to maximise the accuracy of report. Diet recall days were spread across the full week in the total sample as children started the intervention on different days, and covered weekdays and weekend days. All dietary recall data were entered and cross-checked by registered dietitians using the “FoodWorks” dietary analysis software program (FoodWorks 9 Professional, Version 9.0. Brisbane: Xyris Pty Ltd, 2019) which incorporates the New Zealand Food Composition Tables [14]. Dietary intake from the two relevant recalls in each intervention week were adjusted to represent usual intake for that week using the Multiple Source Method (MSM) [26]. Food type was assessed using two methods. Foods were labelled as ‘core’ (foods that form the basis of a healthy diet such as wholegrains, fruit and vegetables) or ‘non-core’ (foods/beverages with lower nutrient-density, such as foods with low concentration of beneficial nutrients per gram and higher fat and sugar content, consumed mainly for pleasure e.g. cakes, cookies, and crisps) based on previous classification systems [27, 28], adjusted as appropriate for the New Zealand diet. Foods were also classified as ‘ultra-processed’ based on the NOVA classification system which groups foods according to the nature, extent, and purposes of the industrial processing that they undergo [29]. To classify individual foods as ultraprocessed, the ‘NUTRITRACK 2018’ database was accessed, which contains a list of all commercially available foods in New Zealand [30]. Further details can be found in a previous paper [16].

### Statistical analyses

Only participants who had complete and valid accelerometry and dietary data for both weeks (extension and restriction) were included. To focus on the impact of sleep loss, only data from those participants who had less sleep on average during the restriction week compared to the extension week were used.

Non-wear time was first reallocated to sedentary, LPA, and MVPA proportionally [31]. Time use compositional data (sleep time, wake after sleep onset (WASO), sedentary time, LPA, and MVPA) were used to calculate isometric log ratios (ilr) for extension and restriction weeks separately. Pivot coordinates of the time use components were used, so that the first ilr referred to the component of interest (e.g. sleep) relative to all other components. Data were in long-form and mixed effects regression models were carried out with dietary intake as the outcome variable, the ilrs as the independent variables, and participant as a random effect. The coefficient of the first ilr represents the average change in dietary intake for a one-unit change in the ilr. Because a one-unit change in ilr is not so meaningful, the coefficient was converted to represent the mean decrease in sleep (48 min, or 8.5%) using methods outlined in Dumuid et al. [11]. This assumes 8.5% of sleep time is reallocated to all other time use components proportionally, which has been shown to be a good approximation [15]. Similarly for the models that investigated sedentary time, LPA, and MVPA, the coefficient was converted to represent the average gain in time in that component. As an exploratory analysis to investigate the potential moderating effect of sex on associations between sleep loss and dietary intake, analyses were also undertaken stratified by sex and reported for a 10% loss in sleep (for comparability). Interaction tests were not undertaken as the study was not powered for these. Confidence intervals (95%) and p-values were calculated and reported. Residuals of all models were plotted and assessed for homoskedasticity and normality.

## Results

**Fig. 1:**
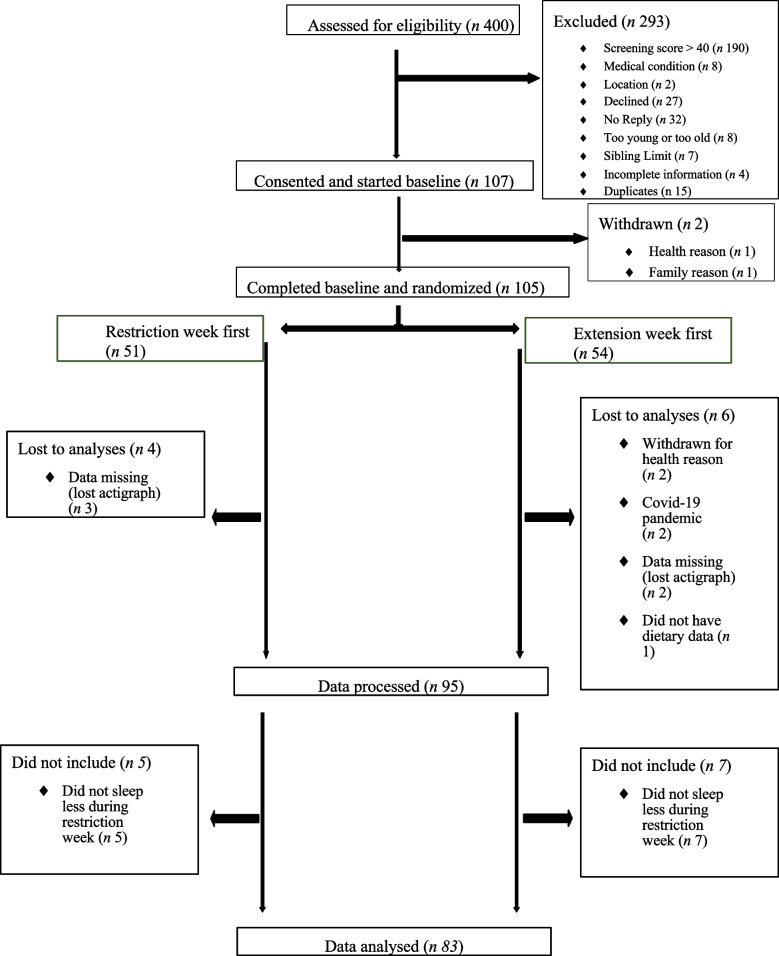
Flow of participants through the study

One hundred and five participants were recruited into the DREAM study. Ninety-six of these participants had complete accelerometry data for both extension and restriction weeks. From these, 84 children were shown, on average, to have sleep loss in the restriction week compared to the extension week, and one of these did not have dietary data for one week. This resulted in 83 participants for this analysis (Fig. 1). Participants were 10 years old on average, 53% were female, and 62% were of a healthy weight. They were mostly of NZ European ethnicity (74%), of medium-to-low deprivation (82%), and 48% of mothers had a university degree (Table 1).

**Table 1:** Demographics, time-use, and dietary intake for those with sleep loss (*n* = 83)

Variable	Descriptive statistic
Age, mean SD years	10.2 (1.4)
Sex, n (%) female	44 (53.0)
BMI z-score, mean (SD)	0.74 (1.18)
Weight status^a^, n (%)	
Normal weight	51 (61.5)
Overweight	18 (21.7)
Obese	14 (16.9)
Ethnicity, n (%)	
New Zealand European	61 (73.5)
Māori	13 (15.7)
Others	9 (10.8)
Area-level deprivation^b^, n (%)	
High (NZDep 8–10)	15 (18.1)
Medium (NZDep 4–7)	33 (39.8)
Low (NZDep 1–3)	35 (42.2)
Maternal education, n (%)	
Secondary school	21 (25.3)
Post-secondary education	18 (21.7)
University degree	40 (48.2)
Time-use in extension week, geometric mean (95% CI) minutes	
Sleep	556 (548, 565)
Sedentary	507 (492, 522)
LPA	280 (270, 290)
MVPA	56 (51, 60)
WASO	16 (13, 21)
Changes in time-use from extension week to restriction week, mean (SD) minutes	
Sleep	-47.9 (30.1)
Sedentary	31.3 (43.7)
LPA	20.8 (31.6)
MVPA	4.2 (14.4)
WASO	-8.5 (21.4)
Dietary intake in extension week, mean (SD)	
Energy intake, kJ	8449 (1454)
Fat intake, g	74.1 (14.4)
Protein intake, g	69.1 (15.5)
Carbohydrate intake, g	257.5 (46.0)
Total sugar intake, g	108.6 (24.8)
Energy intake after 5 pm, kJ	3145 (1158)
UPF, kJ	5731 (1987)
Core foods, kJ	4408 (1808)
Non-core foods, kJ	4960 (2071)

*LPA* light physical activity, *MVPA* moderate-to-vigorous physical activity, *WASO* wake after sleep onset, *UPF* ultra-processed foods

^a^Categories based on the WHO BMI z-score cut-offs; children with underweight <  = -2SD, normal weight > -2SD to + 1SD, overweight >  + 1SD to + 2SD, obesity >  + 2SD

^b^Uses the New Zealand Index of Deprivation 2018 which reflects the extent of material and social deprivation and is used to construct deciles from 1 (least deprived) to 10 (most deprived)

On average, children slept for 9.3 h in the extension week and reduced their sleep by 48 min in the restriction week, as well as reducing their time awake in the night by 8.5 min (Table 1). In the restriction week, children spent the extra time awake being sedentary (31 min more) and more physically active (21 min more LPA and 4.2 min more MVPA). The mean daily activity composition (described by geometric means closed to 1440 min) in the extension week was: sleep = 566 min; WASO = 16.7 min; sedentary = 516 min; LPA = 285 min; and MVPA = 56.5 min. In the extension week, participants consumed just over 8,000 kJ a day, with around 37% of this consumed after 5 pm. Notably, children consumed approximately 68% of their energy as UPF and 59% as non-core foods (Table 1). When participants were mildly sleep deprived they consumed, on average, over 400 kJ more per day, but they consumed over 500 kJ more from UPF and non-core foods compared to when they were well-rested (Table 2).

**Table 2:** Associations between sleep loss and change in dietary intake (*n* = 83)

	Mean difference in dietary intake (SD) between extension and restriction weeks	Mean change in dietary intake for average sleep loss^a^ (95% CI)	*p*-value
Energy intake, kJ	401 (1391)	262 (55, 470)	0.013
Fat intake, g	1.7 (15.4)	1.6 (-0.6, 308)	0.143
Protein intake, g	2.6 (14.3)	2.4 (0.3, 4.5)	0.024
Carbohydrate intake, g	16.5 (45.8)	9.0 (2.1, 15.9)	0.010
Total sugar intake, g	8.6 (24.8)	3.9 (0.2, 7.6)	0.040
Energy intake after 5 pm, kJ	256 (1512)	295 (101, 489)	0.003
UPF, kJ	511 (1872)	281 (-6, 568)	0.055
Core foods, kJ	-18 (1804)	82 (-181, 345)	0.541
Non-core foods, kJ	552 (1993)	341 (43, 638)	0.025

*UPF* ultra-processed foods

^a^Average sleep loss was 48 min (an 8.5% decrease in sleep). These estimates account for corresponding changes in WASO, sedentary time, light physical activity, and moderate-to-vigorous physical activity using compositional analysis

The mean change in daily energy intake for 48 min less sleep time (an average 8.5% decrease in sleep) was 262 kJ (95% CI: 55, 470; *p = *0.013) (Table 2). There was also significantly greater energy intake after 5 pm (295 kJ (101, 489); *p = *0.003) and as non-core foods (341 kJ (43, 638); *p* = 0.025). These estimates represent the effect of sleep change on dietary intake accounting for changes in physical activity and sedentary time.

With less sleep in the restriction week, there were corresponding gains in sedentary time and physical activity. There was no evidence to suggest that the gains in LPA were associated with changes in dietary intake (Table 3). However, a 31 min gain in sedentary time (an average 6.0% increase) was related to increases in energy intake after 5 pm (117 kJ (25, 208); *p* = 0.013) and energy intake from non-core foods (177 kJ (25, 329); *p* = 0.022). In contrast, a 4 min gain in MVPA (an average 7.1% increase) was significantly related to a 75 kJ increase in energy intake (23, 126; *p = *0.004), with this mostly being attributed to a 72 kJ (7, 136; *p = *0.029) increase in core foods. These results account for changes in sleep time but should also be interpreted in the context that these children were mildly sleep deprived.

**Table 3:** Associations between gain in sedentary and physical activity times and change in dietary intake (*n* = 83)

	Mean change in dietary intake for average sedentary time gain^a^ (95% CI)	*p*-value	Mean change in dietary intake for average LPA gain^a^ (95% CI)	*p*-value	Mean change in dietary intake for average MVPA gain^a^ (95% CI)	*p*-value
Energy intake, kJ	64 (-44, 173)	0.245	21 (-93, 136)	0.714	75 (23, 126)	0.004
Fat intake, g	0.3 (-0.8, 1.4)	0.560	0.1 (-1.0, 1.2)	0.848	0.5 (0.0, 1.0)	0.040
Protein intake, g	0.1 (-1.0, 1.2)	0.801	0.7 (-0.4, 1.9)	0.221	0.6 (0.1, 1.1)	0.023
Carbohydrate intake, g	2.4 (-1.1, 6.0)	0.182	1.0 (-2.8, 4.7)	0.608	2.2 (0.5, 3.9)	0.010
Total sugar intake, g	0.8 (-1.1, 2.7)	0.414	1.0 (-1.1, 3.1)	0.340	0.7 (-0.2, 1.6)	0.137
Energy intake after 5 pm, kJ	117 (25, 208)	0.013	16 (-76, 108)	0.734	57 (18, 98)	0.005
UPF, kJ	90 (-61, 242)	0.244	22 (-140, 184)	0.789	66 (-6, 138)	0.073
Core foods, kJ	-32 (-169, 105)	0.648	-3 (-148, 142)	0.968	72 (7, 136)	0.029
Non-core foods, kJ	177 (25, 329)	0.022	-7 (-167, 152)	0.928	47 (-23, 118)	0.191

*LPA* light physical activity, *MVPA* Moderate-to-vigorous physical activity, *UPF* ultra-processed foods

^a^Average sedentary time gain was 31 min (6.0%), LPA gain was 21 min (7.4%), and MVPA gain was 4 min (7.1%). These estimates account for relevant changes in sleep, WASO, sedentary time, light physical activity, and moderate-to-vigorous physical activity using compositional analysis. Note that these changes are in the context of the child being mildly sleep deprived

Females had strong and significant associations between sleep loss and greater energy intake, with significantly higher carbohydrate, total sugar, UPF and non-core food intakes. There were no significant relationships between sleep loss and dietary intake in males and the magnitude of the estimates was substantially lower than in females (Table 4).

**Table 4:** Exploratory analysis of associations between sleep loss and change in dietary intake by sex (*n* = 83)

	Mean change in dietary intake for 10% sleep loss^a^ (95% CI)	
	Female	Male
n	44	39
Energy intake, kJ	**512 (146, 877)**	85 (-217, 388)
Fat intake, g	2.8 (-0.8, 6.4)	0.4 (-3.1, 3.9)
Protein intake, g	2.7 (-0.7, 6.1)	2.4 (-1.0, 5.8)
Carbohydrate intake, g	**19 (7, 32)**	3 (-7, 13)
Total sugar intake, g	**10 (3, 18)**	0 (-5, 5)
Energy intake after 5 pm, kJ	**417 (120, 713)**	224 (-98, 546)
UPF, kJ	**597 (149, 1046)**	67 (-414, 548)
Core foods, kJ	209 (-276, 693)	-54 (-429, 321)
Non-core foods, kJ	**629 (122, 1135)**	178 (-291, 647)

*UPF* ultra-processed foods

^a^A 10% sleep loss was 56 min for females and 55 min for males. These estimates account for corresponding changes in WASO, sedentary time, light physical activity, and moderate-to-vigorous physical activity using compositional analysis

## Discussion

Our data show that even a relatively mild decrease in sleep over one week was associated with significantly increased energy intake in children, especially of non-core foods. Importantly, this elevation in intake was independent of accompanying increases in sedentary time and physical activity that occurred with sleep restriction. Interestingly, changes in the activity composition of the day were reflected differently in terms of diet; children who tended to increase their levels of MVPA, albeit by a small amount, reported a greater energy intake from core foods. By contrast, children who showed larger increases in sedentary time ate greater amounts of energy from non-core foods and during the evening hours.

To date, few interventions have examined the effect of changing sleep on dietary intake in children, and none that have simultaneously accounted for the corresponding changes in other components of 24-h time use that must occur when sleep is altered. Previous mechanistic studies in preschool [32], primary (middle) school aged [33] children, and adolescents [34] have also indicated that short-term reductions in sleep over one to seven nights results in significantly increased energy intakes. Few studies have examined whether improvements, rather than reductions, in sleep reduce energy intake, either short- or longer-term in children. Hart et al. [35] reported no change in caloric intake following a two-month intervention in 8–11 year old children with poor sleep, although studies in adults indicate that such benefits do occur [36, 37].

Similarly, little experimental research has examined which components of the diet might change in response to altered sleep. In the current study, we observed differences in macronutrient composition, which supports the findings of some [32, 34], but not all [33, 35, 38] previous studies, although it could be argued that macronutrient differences are unlikely unless energy intake has also changed significantly. Although a reasonable body of observational literature indicates that short sleep is associated with lower dietary quality [39, 40], the bulk of this research is cross-sectional in nature and thus unable to determine causality. Our finding that the increased caloric intake was mostly due to increases in non-core or ultraprocessed foods rather than core foods, as well as sweetened beverages [16] supports existing experimental research in adolescents [34, 38] and adds support to these observational findings [39, 40].

Although previous experimental work has examined how changes in an individual time use behaviour (e.g. sleep or physical activity or sedentary behaviour) influences diet [41–43], no studies appear to have used compositional analyses to investigate how these behaviours interact to influence what children eat. Our data showed that the increased sedentary time that occurred with sleep restriction was associated with more energy from non-core foods, while the increased time in MVPA was also associated with more energy, but from core foods. Overall, the magnitude of changes in energy intake suggest that the impact of sleep deprivation on lower diet quality is mostly from changing sleep, rather than any increases in physical activity. Thus, sleep appears to be the most influential time use component on dietary intake, indicating that interventions that focus on improving sleep may be an appropriate way of also improving diet. In our study, no dietary advice was provided, yet substantial differences in dietary intake were observed, most markedly in girls – although this was an exploratory analysis. While some observational studies have shown differences in sleep and diet between girls and boys [39], this does not appear to have been explored in experimental studies. Similar results for the impact of sleep on diet have been reported in adult studies [44]. Given current concern about the quality of children’s diets and known difficulties in improving dietary intake [44], altering sleep may offer an intriguing ‘stealth’ mechanism for improving dietary quality in children that requires further work.

Our randomised controlled trial has many strengths. DREAM was a carefully controlled mechanistic study, trying to determine whether it was changes in dietary intake, or alterations in physical activity and sedentary time that might explain why children who do not get enough sleep have a stronger risk of obesity [1, 2]. First, our study design (crossover) meant that each child could act as their own control, thus reducing the potential for unknown confounders or differences between participants to influence outcomes. Second, our drop-out rate was much lower than anticipated, such that we had large numbers of children who were able to adhere to the protocol and alter their sleep accordingly. This allowed us to use the most powerful type of data (experimental) to truly show what happens to activity levels and dietary intake when sleep is altered. Third, we had objective measures of all aspects of energy expenditure, using actigraphy to measure 24-h time use. This negated any issues with trying to match different data sources (e.g. using questionnaires to measure sleep and actigraphy for daytime physical activity) to ensure complete data for individuals. It also allowed us to examine our research questions using appropriate analyses to account for the compositional nature of the data [31]. Fourth, we used wearable cameras as part of our dietary assessment, to remind children and parents about potentially forgotten foods during the 24-h recall process [17]. Finally, we undertook analyses that looked at both sides of the energy balance equation (i.e. energy in vs energy out) which meant we could adjust for each accordingly.

Our study also has some limitations. As DREAM was a mechanistic study rather than a behavioural sleep intervention per se, we cannot answer whether changing sleep will make long-term changes to dietary intake and/or levels of sedentary time or physical activity. It may be that children adapt over time when they experience chronic rather than acute mild sleep deprivation. Our sample had some diversity, but was predominantly European and relatively well-educated, with a lower proportion of children from homes with greater deprivation than is expected nationally (22% compared with 30%), which may limit extrapolation to other groups. There are also well-known limitations inherent with dietary assessment, particularly around under-reporting of energy intake. We tried to overcome this issue as much as possible by having both child and parent present and by using wearable camera images as memory prompts. While we did not formally evaluate whether these images produced a significant improvement in the quality of our recall data as has been shown elsewhere [45], those completing the diet recalls with our families indicated that it was not uncommon for foods and beverages to be added to the recalls upon examination of the images.

## Conclusion

Our data show that when children sleep less they increase their energy intake, mostly from non-core foods, by amounts that could explain the strong relationship observed between reduced sleep and obesity in children. Importantly, these differences in dietary intake were observed after controlling for any changes in energy intake as a result of changes in activity levels during the day. These data suggest that interventions focusing on improving sleep may be beneficial for improving both dietary intake and weight status in children.

### Supplementary Information

Supplementary Material 1.

## Data Availability

Data described in the manuscript, code book, and analytic code will be made available upon request pending application and approval.

## Abbreviations

BMI-z: Body mass index z-score
CoDA: Compositional data analysis
DREAM: Daily Rest Eating and Activity Monitoring study
LPA: Light physical activity
MVPA: Moderate to vigorous physical activity
PA: Physical activity
RCT: Randomized controlled trial
SD: Standard deviation
SPT: Sleep period time
TST: Total Sleep Time
WASO: Wake after sleep onset
WHO: World Health Organization
NZ: New Zealand
NZDep: New Zealand Deprivation Index
